# Longitudinal changes of mental health problems in children and adolescents treated in a primary care-based health-coaching programme – results of the PrimA-QuO cohort study

**DOI:** 10.1186/s12875-022-01780-1

**Published:** 2022-08-22

**Authors:** Siona Decke, Karina Hamacher, Martin Lang, Otto Laub, Lars Schwettmann, Ralf Strobl, Eva Grill

**Affiliations:** 1 Institute for Medical Information Processing, Biometry, and Epidemiology - IBE, LMU Munich, Munich, Germany; 2 Pettenkofer School of Public Health, Munich, Germany; 3 BKK Vertragsarbeitsgemeinschaft Bayern, Munich, Germany; 4 Berufsverband der Kinder- und Jugendärzte (BVKJ) e.V., Cologne, Germany; 5 PaedNetz Bayern e.V., Munich, Germany; 6 Institute of Health Economics and Health Care Management (IGM), Helmholtz Zentrum München - German Research Center for Environmental Health (GmbH), Neuherberg, Germany; 7 Department of Economics, Martin Luther University Halle-Wittenberg, Halle (Saale), Germany; 8 German Centre for Vertigo and Balance Disorders, University Hospital, LMU Munich, Munich, Germany

**Keywords:** Mental Health Problems, Children and Adolescents, Paediatrician, Health Coaching Programme, Cohort Study

## Abstract

**Background:**

In Germany, 19.1% of boys and 14.5% of girls are affected by mental health problems (MHP). Paediatricians are usually the first in line to be contacted but they often do not feel adequately trained to diagnose and treat MHP in primary care. A major statutory health insurance fund introduced a health coaching (HC) programme to strengthen primary care consultation for MHP. The HC includes a training concept for paediatricians, standardised guidelines for actions and additional payments. The aim of this study was to investigate the potential effects of the HC programme on the change of MHP in children and adolescents.

**Methods:**

A prospective cohort study was conducted in Bavaria, Germany, in 2018 and 2019. Data were collected at 2 points 1 year apart using an online questionnaire. Parents of patients with developmental disorder of speech and language, head/abdominal pain, conduct disorder or non-organic enuresis were approached by their health insurance. Families treated according to the HC programme form the intervention group while all others serve as controls. MHP was assessed using the Strengths and Difficulties Questionnaire (SDQ) as a child self-assessment (SDQ-S)/or external assessment by parents (SDQ-P). Determinants of SDQ total score were analysed using linear mixed effects models.

**Results:**

Cross-sectional (*n* = 1090) and longitudinal analyses (*n* = 599) were performed. At baseline, a total of 23.5% had an SDQ total score “at risk” (SDQ-S > 15/SDQ-P > 13). There were no significant differences between intervention and controls. After full adjustment for all potential confounders, higher SDQ scores indicating more problems were significantly associated with male sex (2.000, *p* < 0.001) whereas a high parental education level was significantly associated with decreased SDQ scores (-2.127, *p* =0.034). There was a significant improvement in the control group over time (-0.814, *p* = 0.001) while the SDQ scores in the intervention group remained stable (-0.012, *p* = 0.020).

**Conclusion:**

This evaluation of the HC programme could not prove a clinically relevant intervention’s effect on the MHP developmental course. Nevertheless, (HC) paediatricians have crucial potential to improve the care of MHP patients. Targeting families with less access to support measures might help reduce the burden of MHP and be a step towards continuous improvement of care.

**Supplementary Information:**

The online version contains supplementary material available at 10.1186/s12875-022-01780-1.

## Background

Mental health is an important prerequisite for happiness, quality of life and wellbeing [1]. Mental health problems (MHP) of children and adolescents can constitute health impairments with major implications regarding daily and social functioning, performance at school and later professional development [2, 3]. Moreover, these conditions can cause economic burdens for families and healthcare systems [4–6]. MHP of children and adolescents are therefore regarded as a highly relevant public health issue in all countries of the world [7, 8]. According to the German Child and Youth Health Survey (KiGGS), 19.1% of boys and 14.5% of girls aged 3–17 years are affected by MPH in Germany [9]. Among MHP, developmental disorders (17%) and conduct disorders (11%) were the most common conditions seen in paediatric care [10].

Effective and evidence-based therapies for children and adolescents with MHP such as cognitive-behavioural therapy have been established [11–13]. However, only 30% of minors with MHP in Germany [14] and other industrialised countries [15, 16] have access to appropriate medical care. Waiting time, settings that fail to meet parents’ and children’s needs, long travelling distances and lack of intersectoral communication and treatment have been identified as the most relevant barriers to impede or delay timely access to professional assessment and therapy [7]. In Germany, paediatricians in primary care are often either the first in line to be consulted for MHP [14] or they detect MHP during the developmental examinations that are routinely and regularly carried out [17]. Yet, it has been shown that many primary care paediatricians do not feel adequately trained and therefore tend to underdiagnose and undertreat MHP patients in primary care [18, 19]. Enhanced training has been shown to be a promising intervention to strengthen and support the paediatricians’ skills in the detection of MHP and in the delivery of simple interventions [20–23].

Against this background, a major German statutory health insurance fund (“Betriebskrankenkassen Landesverband Bayern”—BKK-LV Bayern) in cooperation^1^ with a professional association of paediatricians (^“^Berufsverband der Kinder- und Jugendärzte” – BVKJ e. V.) has introduced a programme for their insured members that targets primary care paediatricians (Health Coaching—HC) in 2013 [24]. The programme development started in 2011 and was based on mutual consultations of medical stakeholders. The HC programme includes a training concept for paediatricians, standardised guidelines for actions for 16 specific mental health conditions and additional fees for paediatricians who complete this training and treat children and adolescents with MPH according to the guidelines.^2^ The BKK funds provide an additional budget for the use of the standardised guidelines for 16 defined MHP beyond the conventional statutory health insurance (SHI) service spectrum regarding social-paediatric-oriented in-depth counselling, discussion and/or clarification as well as continuing social-paediatric-oriented care^3^ [25, 26]. The HC aims to provide improved integrative care for children and adolescents with MHP in paediatric practice by training paediatricians in the detection and treatment of MHP. Furthermore, the programme tries to impart self-management skills to the children and their parents and purposefully inform them about the various care services available. The basic programme’s principles are participation, patient orientation and strengthening of existing resources. The underlying model of the HC and its intervention components is the International Classification of Functioning, Disability and Health in the version for children and adolescents (ICF-CY) [27]. ICF-CY is a complex classification standard. It takes developmental peculiarities and special living environments of children and young people into account and provides a framework and common language and for formulating and planning support, therapies and treatment goals. Prior to the present analysis, an expert interview with the programme developers was performed (not published). In addition, the implementation of the programme in paediatric practice and the perception of patients and families involved was assessed by Decke et al. [28]. Among the interviewed paediatricians, 3 paediatricians stated that they had also been involved in contract negotiations and in HC development, which enriched the findings too. The interviews revealed that the programme is well received by paediatricians, patients and their families [28]. However, the HC programme has not been systematically evaluated yet. It is hypothesised that the HC is an effective primary care programme improving patients’ and their families’ health outcomes. The objective of this study was therefore to investigate the potential effects of the HC programme on the change of MHP in children and adolescents. Medical utilisation and cost effects were examined by Marijic et al. [29]*.* A detailed description of the study objectives, the study design and the methodological procedure can be found in the study protocol [30].

The term "children" includes children and adolescents aged 0–17 years.

## Methods

### Study design

A prospective cohort study was conducted in Bavaria, Germany, from January 2018 up to November 2019. The collection of data was performed using an online questionnaire. Data were collected at 2 time points 1 year apart.

### Intervention

**Fig. 1:**
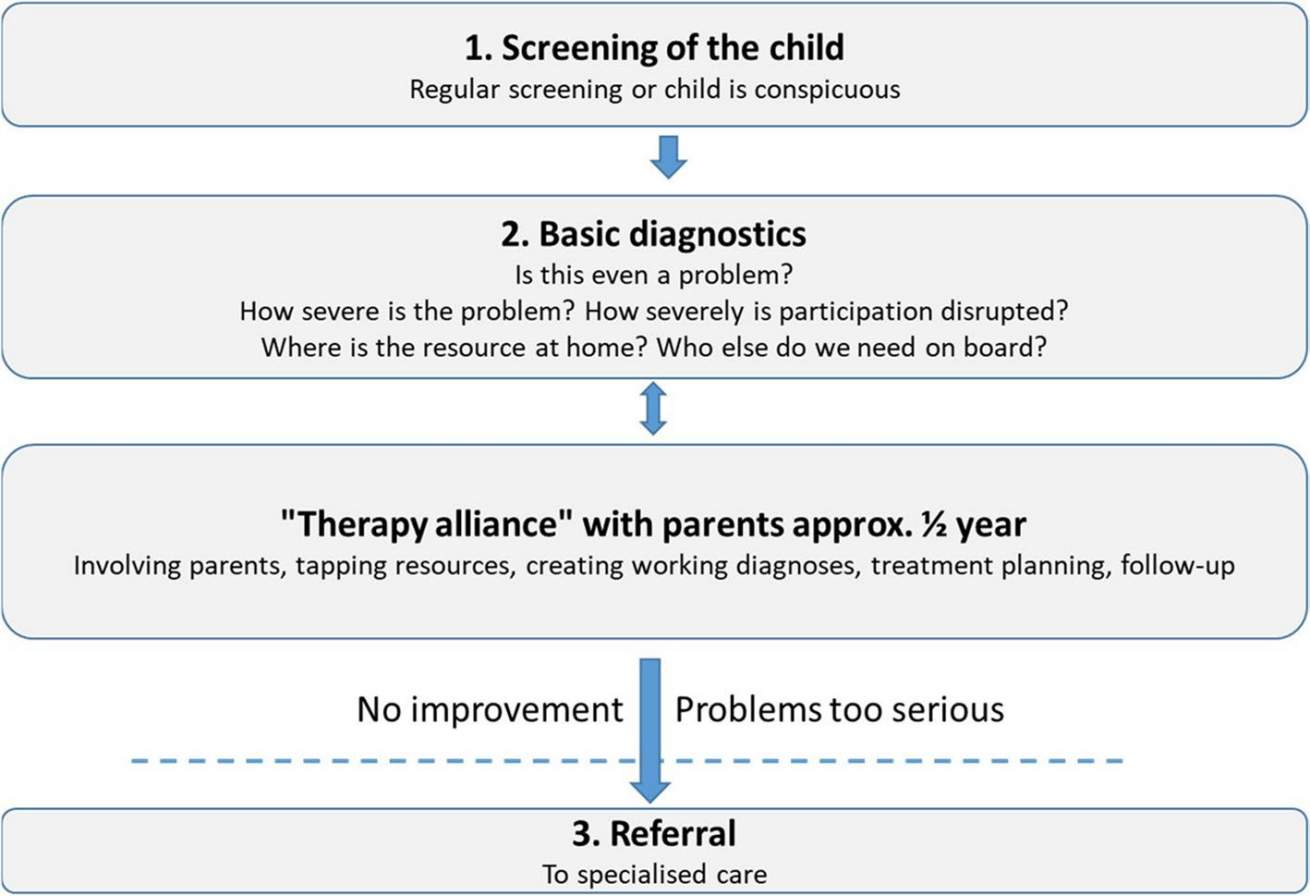
Three steps model of social-paediatric diagnostics

The HC is mainly implemented in Bavaria, one of the largest federal states in Germany with a total population of 13 million people. The HC has been available nationwide since October 2015. More than 700 paediatricians in Bavaria and more than 2100 nationwide are currently qualified to participate in the HC programme and approximately 36,000 children with MHP have been treated according to the programme. The HC includes a training concept for paediatricians based on a dual training participation, standardised action guidelines for 16 MHP (e. g. developmental disorder of speech and language, enuresis, head and abdominal pain) and additional fees for paediatricians who undergo this specific training and demonstrably act according to the guidelines as shown in Fig. 1. With the HC, an additional 15 euros per 10 min can be billed up to a cap of 180 min per child (in addition to the SHI standard care).

**Fig. 2:**
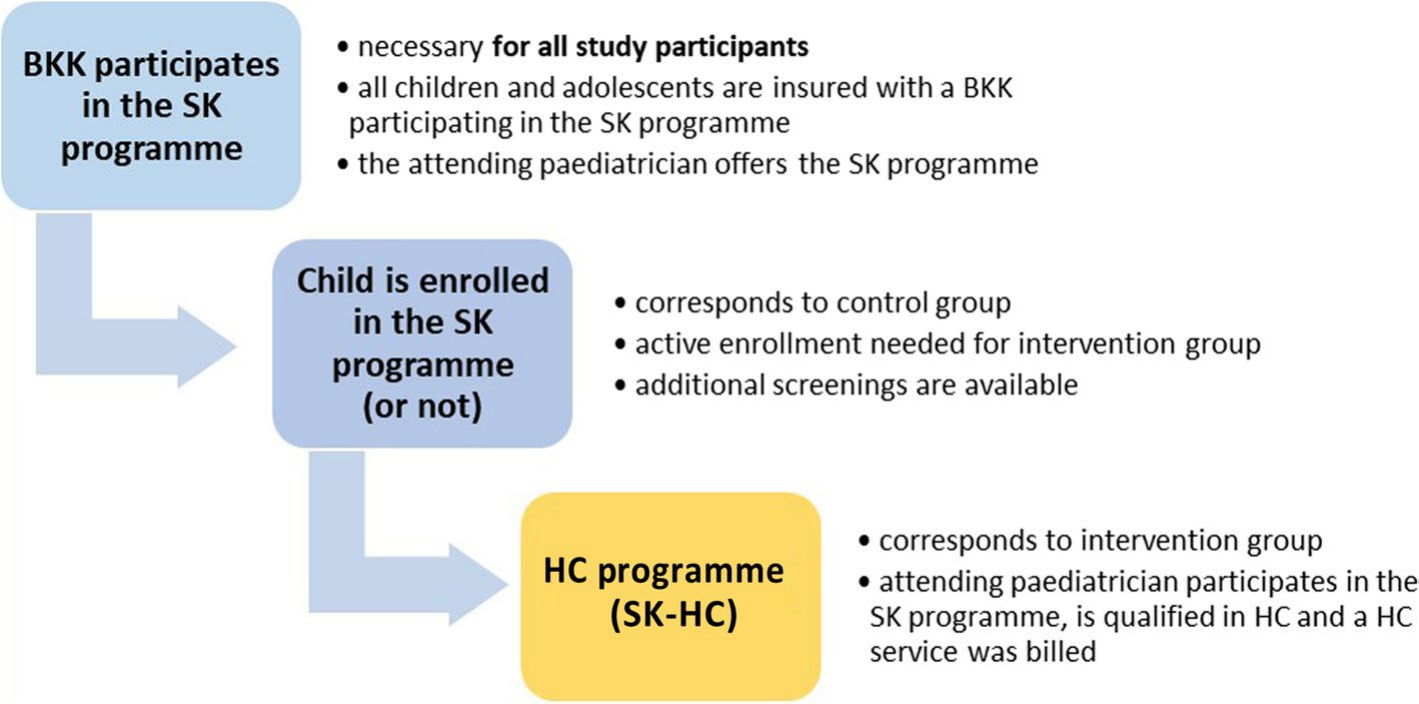
Overview of the intervention (SK-HC) and control group (enrolled in SK or not)

The BKK programme “STARKE KIDS” (SK) forms the basis of the HC. With the SK programme, further developmental check-ups are available for children enrolled in the programme. In addition, the HC programme can be offered to children and adolescents with MHP as shown in Fig. 2. To implement the HC programme, the paediatrician must participate in the SK programme and complete a dual HC training participation while the child needs to be enrolled in the SK programme so that HC services are billable. The paediatricians’ participation in two HC training courses is mandatory for billing the programme. More details are given in the Supplement.

### Setting and sample

Parents of patients were included if at least 1 of their children (up to 17 years old) had been diagnosed with 1 (or more) of the 4 most common MHP diagnoses indicated by ICD codes^4^ (10^th^ revision), if they were insured with the BKK health insurance company and had at least 1 consultation at an office-based paediatrician in Bavaria, Germany, due to a MHP of their child/children in the last 6 months. Included diagnoses were 1) developmental disorder of speech and language (ICD Codes: F80.0-F80.9), 2) head and abdominal pain (somatoform) (G44.2, G43.0, G43.1, F45.4, R10.4), 3) conduct disorder (F68.8, F91.0–92.9, F94.0–95.9, F98.3-F98.9) and 4) non-organic enuresis (F98.0) [31].

All children had to be insured with a health insurance fund participating in the SK to enable the retrieval of performance data. With the SK programme, enhanced screenings for minors throughout Germany are available.^5^ Further information is given in the additional file.

Children in the intervention group are SK participants and were treated due to 1 of the MHP included according to the HC programme. Hence, they were treated by a paediatrician with HC qualification and a HC service had been billed.

Children in the control group did not necessarily have to be enrolled in the SK programme themselves (see Fig. 2) and could therefore be SK or non-SK participants. Controls were treated according to 1 of the included MHP by a paediatrician who offers the SK programme, but no HC services had been billed.

All participants who met the inclusion criteria were identified by the BKK based on the billing data. Billing data were available with an average delay of 6 months. Eligible parents were invited by their health insurance company by letter and provided with a link to the online questionnaire. After 1 year, all participants received their follow-up invitation and login details per email. The questionnaire was answered by the parents or by the children themselves (if aged 11 or older).

All participants invited received age-appropriate study information with the possibility to contact the study centre in case of questions. Participants were informed about the data protection measures and signed an informed consent form before starting the questionnaire. Informed consent was obtained from the parents and the child if aged 6 years and older. Participation was voluntary. Participants also received information about the confidentiality of the questionnaire and the opportunity to stop participation at any time without giving any justification. Families were offered a small monetary compensation for their participation.

Approval from the Ethics Committee (registration number 17–497) and the Data Protection Officer of the Medical Faculty of the Ludwig-Maximilians-Universität Munich (LMU) was obtained prior to the start of the study. All data protection measures fulfilled the European and national data protection regulations (EU-DSGVO and BDSG) [32]. The STROBE (STrengthening the Reporting of OBservational studies in Epidemiology) checklist was used to support the complete and transparent reporting of our research.

### Variables and measurements

Outcome of interest was the change of the child’s MHP. The assessment of MHP in our sample was carried out using the Strengths and Difficulties Questionnaire (SDQ) [33, 34]. The SDQ is a screening instrument of 25 items that contains 5 different subscales measuring 1) emotional symptoms, 2) conduct problems, 3) hyperactivity-inattention, 4) peer relationship problems and 5) pro-social behaviour. Each of the SDQ items is scored on a 3-point Likert scale with 0 = not true, 1 = somewhat true or 2 = certainly true. Higher scores indicate greater problems, except for pro-social behaviour, where a higher score indicates more positive behaviour. A total difficulties score (range 0–40) can be obtained by summing the scores of the subscales 1–4. Higher values in the total score or in the 4 problem scales indicate a higher symptom burden, whereas higher values in the strength scale 5) pro-social behaviour indicate an increase in pro-social behaviour. Moreover, using the SDQ impact supplement, the study provides information on psychosocial impairment following child and adolescent MHP. The SDQ is available as a parental (SDQ-P) or self-assessment version (SDQ-S) for children aged 11 years or older. In agreement with this age cut, the SDQ-S version was completed by the child, or the proxy version (SDQ-P) was completed by the parents for younger children (< 11 years of age). Because sample size was too small in diagnostic subgroups, parental and self-assessment of the SDQ were combined for subsequent analyses. In accordance with German normative data [35, 36] and the cut-offs used in KiGGS [9], a SDQ score of > 13 (SDQ-P) and > 15 (SDQ-S), respectively, were considered as indicative of a mental health problem.

Sociodemographic data, namely age and sex of the child as well as age, sex and educational level of parents were collected at baseline. Age of the child was categorised (< 3 years of age, 3–5, 6–8, 9–11, 12–14 and 15 years or older) according to the KiGGS study [9]. The highest educational level of both parents was used and categorised into low (no qualification or secondary school), medium (intermediate school, no high school graduation) and high (high school or university graduation). The questionnaire was presented in German. Therefore, it must be assumed that families with a migrant background are not a representative sample of all migrant families living in Germany. This is why we decided not to report migrant background. The parents’ income was not assessed in our study.

### Statistical analysis

We report means and standard deviations for continuous variables as well as absolute frequencies and percentages for categorical variables. We compared SDQ scores of children with MHP receiving HC treatment (HC group) to children with the same diagnosis receiving standard paediatric care (control group). We compared the change in the scores (follow-up minus baseline score) in both groups as well as the change in MHP subgroups. The linear trend for each subject was visualised. P-values for differences in characteristics were based on Chi-square tests for categorical and Kruskal–Wallis tests for continuous variables. Significance level was set at *p* = 0.05.

Determinants of SDQ total score were analysed using linear mixed effects models (LME). LME allow to model the longitudinal relationship of different risk factors on an outcome by taking correlation structure of repeated measurements into account [37]. Random subject-specific intercepts were included to adjust for variance in the outcome between the subjects. Interaction terms of times and the respective risk factors were included to model the effect on the change of the SDQ values over time. According to the SDQ authors, a minimal difference of more than 2 points in the SDQ total score over time is considered relevant. Model fit was assessed by the Akaike Information Criterion (AIC) with lower values indicating better fit. Covariate selection was based on the literature indicating differences in MPH according to the age and sex of the child, migrant background and educational level of the parents and differences depending on which MHP is involved [38–41]. Therefore, age and gender of the child, intervention group (HC vs. control), parental educational level and the 4 indications (head and abdominal pain, developmental disorder of speech and language, enuresis and conduct disorder) were included in each model. We report an unadjusted model with time as the only covariate, a model with age and sex, and a fully adjusted model.

Inverse Probability of Treatment Weighting was calculated and introduced into each of the models to compensate for the lack of randomisation in group allocation [42, 43]. Only randomisation guarantees an equal distribution of all known and unknown patient characteristics in an intervention and a control group and thus allows causal conclusions about the treatment effects of therapy. When randomised controlled trials are not feasible, studies are at risk for treatment selection bias. Propensity scores minimise this bias by balancing the known confounders between treatment groups. The propensity score (PS) is defined as the probability of a patient receiving the therapy to be tested. The PS is estimated in a first step. In a second step, the actual therapy effect of interest is estimated including the PS.

All statistical analyses were carried out using SAS (Version 9.4, SAS Institute, Inc., Cary, NC, USA).

### Sensitivity analysis

Results might be sensitive to categorisation of the outcome. Internationally, varying SDQ cut-offs are available, which impedes comparability [44]. In Germany, a SDQ total score of > 13 (SDQ-P) and > 15 (SDQ-S), respectively, are considered as indicative of a mental health problem (“at risk”). Our main analysis focused on the change in the total score as recommended by the SDQ authors [45]. Nevertheless, we also modelled the change in the SDQ cut-offs “not at risk” (SDQ-P ≤ 13/SDQ-S ≤ 15) and “at risk” (> 13/ > 15). Absolute frequencies and percentages in the SDQ cut-offs for both groups were reported. *P*-values for differences in the change paths “improvement” (at risk at baseline and not at risk at follow-up), “deterioration” (not at risk at baseline and at risk at follow-up) as well as “no change” (still not at risk or still at risk) were based on Chi-square tests (significance level: *p* = 0.05). In addition, the age of the child was introduced as a continuous instead of a categorised variable. In the main analyses, SDQ-P and SDQ-S were combined for subsequent analyses due to the small number of children completing the self-assessment version. In the assessment of externalising problems such as hyperactivity or conduct disorder, the parents’ judgement is considered valid. With regard to emotional problems, especially in adolescents, the self-assessment is considered as more sensitive [3]. We therefore decided to model both, the change in SDQ subscales and the change in SDQ-P and SDQ-S additionally.

## Results

The overall response rate at baseline was 17% and 56% at follow-up. More information is given in the supplement.

### Baseline characteristics

**Fig. 3:**
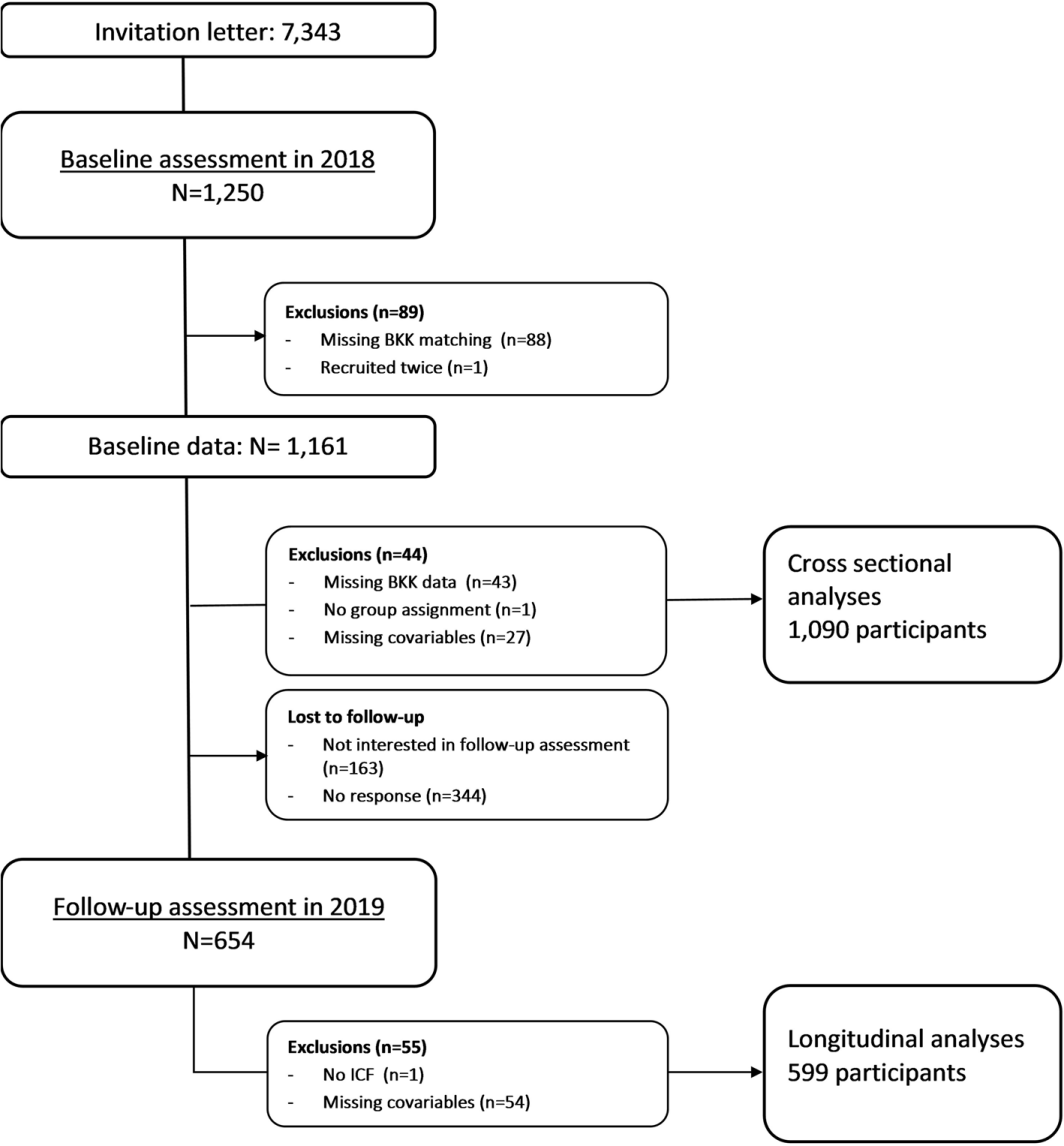
Flow chart of the study population

Overall, 1090 children and their parents were included at baseline. A flow chart is shown in Fig. 3.

The questionnaire was mainly answered by mothers (80.3%). The number of children per family ranged from 1 to 5. Boys represented 60.2% of the participants. The children’s mean age was 6.9 years (SD 3.3). Characteristics of the participants stratified by intervention are shown in Table 1. About ^1^/_3_ (30.6%, *n* = 333) of the children received HC treatment. Conduct disorder (29.7 vs. 21.5%) and enuresis (17.1 vs. 4.6%) were more frequent in the intervention group, while developmental disorders of speech and language (57.3 vs. 41.1%) and abdominal pain (22.2 vs. 16.5%) were more frequent in the control group. The SDQ-P was completed for 922 children aged up to 11 years. 168 minors completed the self-assessment version (SDQ-S). The overall SDQ at baseline was 8.4 (SD 5.7) and 11.1 (SD 6.4), respectively. A total of 23.5% had an “at risk” score (SDQ-P > 13: 23.2%, SDQ-S > 15: 25%) at baseline. There were no significant differences in the SDQ assessment between intervention and control group.

**Table 1:** Characteristics of the study population by intervention and control group at baseline

	**Total**	**HC**	**Control**	***p*-Value**^**+**^
Age of the child^b^				
0–2	58 (5.3)	15 (4.5)	43 (5.7)	0.643
3–5	389 (35.7)	120 (36.0)	269 (35.5)	
6–8	334 (30.6)	97 (29.1)	237 (31.3)	
9–11	192 (17.6)	67 (20.1)	125 (16.5)	
12–14	91 (8.4)	28 (8.4)	63 (8.3)	
15–17	26 (2.4)	6 (1.8)	20 (2	
Boys^b^	656 (60.2)	205 (61.6)	451 (59.6)	0.538
Age of the mother in years^a^	38.1 (5.2)	38.3 (4.9)	38.0 (5.4)	0.637
Age of the father in years^a^	41.4 (6.2)	41.4 (6.1)	40.9 (6.0)	0.413
Educational level of parents^b^				
high	562 (51.6)	175 (52.6)	387 (51.2)	0.808
middle	424 (38.9)	125 (37.5)	299 (39.6)	
low	103 (9.5)	33 (9.9)	70 (9.3)	
MHP diagnosis^b^				
head/abdominal pain	223 (20.5)	55 (16.5)	168 (22.2)	0.032
speech disorder	571 (52.4)	137 (41.1)	434 (57.3)	<0.001
conduct disorder	262 (24.0)	99 (29.7)	163 (21.5)	0.004
enuresis	92 (8.4)	57 (17.1)	35 (4.6)	<0.001
Parental assessment				
SDQ Score (0–40)^a^	8.4 (5.7)	8.5 (5.8)	8.4 (5.6)	0.970
SDQ Score "at risk"^b^	214 (23.2)	64 (22.4)	150 (23.6)	0.688
Impact Score (0–10)^a^	0.6 (1.3)	0.6 (1.4)	0.5 (1.3)	0.247
Self-assessment of the child				
SDQ score (0–40)^a^	11.1 (6.4)	10.9 (6.2)	11.1 (6.5)	0.918
SDQ score "at risk"^b^	42 (25.0)	11 (23.4)	31 (25.6)	0.766
Impact score (0–10)^a^	1.2 (2.0)	1.1 (1.8)	1.2 (2.1)	0.649

^a^mean (standard deviation) ^b^n (%)

^+^Chi-square test for categorical variables, Kruskal–Wallis test for continuous variables

N: Total = 1090 (HC = 333/ Control = 757)

SDQ Parental Assessment: *N* = 922 (HC = 286/ Control = 636)

SDQ Self-Assessment: *N* = 168 (HC = 47/ Control = 121)

### Longitudinal analyses

The follow-up questionnaire was answered by 654 participants. After excluding 55 participants with missing values in the outcome of interest (SDQ) there were no further exclusions necessary due to missing data in basic covariables. In total, 599 participants had sufficiently complete data to be included in the longitudinal analyses as shown in the flow chart (Fig. 3).

During the follow-up, there were no significant differences between the two groups neither in the change of the SDQ total score, nor in the change in SDQ items. The individual change in SDQ total score (SDQ-P and SDQ-S combined) by intervention and control group was visualised (Supplementary Fig. 4). There were no remarkable changes detectable. The overall change in the SDQ total score by intervention and control group as well as the change by diagnosis group are shown in Table 2. There was a slight decrease (-0.6, SD 4.1) in the control group, which was not significant (*p* = 0.065). The highest scores (10.6, SD 5.7) were observed in children with conduct disorder, but there were no significant differences between intervention and control group (*p* = 0.559).

**Table 2:** Change in SDQ total score by diagnosis subgroup

	**Total**	**HC**	**Control**	***p*-Value**^**+**^
SDQ total score at baseline^a^	9.1 (6.0)	9.2 (6.2)	9.0 (6.0)	0.804
SDQ total score at follow-up^a^	8.6 (5.7)	9.2 (5.8)	8.4 (5.7)	0.065
Change in SDQ total score^a^	-0.4 (4.2)	-0.0 (4.4)	-0.6 (4.1)	0.110
Change by diagnosis subgroup^a^				
(1) head/abdominal pain	7.4 (4.7)	7.8 (4.4)	7.3 (4.8)	0.460
	-0.4 (3.6)	-0.0 (3.1)	-0.6 (3.8)	0.713
(2) speech disorder	8.2 (5.8)	8.3 (5.4)	8.1 (5.9)	0.490
	-0.2 (4.1)	0.1 (3.9)	-0.3 (4.1)	0.238
(3) conduct disorder	10.6 (5.7)	10.7 (5.9)	10.5 (5.6)	0.559
	-0.8 (4.7)	-0.0 (5.1)	-1.3 (4.4)	0.113
(4) enuresis	8.9 (5.9)	9.7 (6.2)	8.0 (5.6)	0.367
	-0.7 (4.7)	-0.2 (4.9)	-1.3 (4.4)	0.588

^a^mean (standard deviation); SDQ-P and SDQ-S combined

^+^Kruskal–Wallis test for continuous variables

Total: *n* = 599 participants (176 HC/423 Control); per MHP diagnosis: Head/abdominal pain: *n* = 104 (24 HC/80 Control); Speech disorder: *n* = 330 (73 HC/257 Control); Conduct disorder: *n* = 145 (57 HC/88 Control), Enuresis: *n* = 51 (28 HC/23 Control)

### Linear mixed effects model

Results from the unadjusted and adjusted models of the SDQ total score are shown in Table 3.

**Table 3:** Longitudinal modelling on SDQ total scores

	Model 1: Unadjusted			Model 2: Adjusted for age and sex			Model 3: Fully adjusted		
Variables	ß-Coefficient	Std Error	*p*-Value	ß-Coefficient	Std Error	*p*-Value	ß-Coefficient	Std Error	*p*-Value
Intercept	8.961	0.290	<0 .001	7.905	0.597	< 0.001	11.376	2.345	< 0.001
Intervention (Ref = control)	0.484	0.478	0.312	0.501	0.464	0.283	-0.237	0.501	0.636
Time (Ref = baseline)	-0.414	0.172	0.017	-0.414	0.175	0.017	-0.814	0.242	0.001
Interaction of intervention and time							0.802	0.344	0.020
Age of the child in years (Ref = 0–2)									
3–5				-0.910	1.013	0.370	-1.060	1.011	0.295
6–8				-0.683	1.033	0.509	-0.893	1.036	0.389
9–11				0.861	1.085	0.428	0.388	1.078	0.719
12–14				1.183	1.222	0.334	0.252	1.222	0.837
15–17				2.781	2.359	0.239	1.533	2.344	0.513
Sex (Ref = female)				2.188	0.451	<0.001	2.000	0.450	< 0.001
Head/abdominal pain							-1.098	1.021	0.283
Speech and language							-0.465	0.936	0.619
Conduct disorder							1.778	0.940	0.060
Enuresis							0.254	1.060	0.811
High educational level (Ref = low)							-2.127	1.000	0.034
Intermediate educational level (Ref = low)							-1.792	1.020	0.079
Random intercept	24.162			22.544			21.650		
Residual variance	5.254			5.255			5.213		
AIC	7316.4			7269.7			7232.2		

The β estimates the change in the dependent variable SDQ total score per unit of increase of continuous predictors or in the yes versus no group for binary predictors. SDQ-S and SDQ-P were combined

Negative β-coefficients represent a decrease in SDQ total scores per unit of increase of continuous predictors or in the yes versus no group for binary predictors

Random effects model adjusted for gender, age, educational level of the parents and diagnosis of the child with inverse probability of treatment weighting (IPTW) and random intercept (*n* = 599)

In the unadjusted model, time (-0.414, *p* = 0.017) but not HC treatment (0.484, *p* = 0.312) were associated with lower SDQ scores. After full adjustment for all potential confounders, higher SDQ scores were significantly associated with male sex (2.000, *p* < 0.001). A higher age (15–17 years) was associated with higher SDQ scores, but the effect was not significant (1.533, *p* = 0.513). A high educational level of the parents was significantly associated with lower SDQ scores (-2.127, *p* = 0.034). There was a significant improvement in the control group over time (-0.814, *p* = 0.001). SDQ values in the intervention group remained stable (-0.012 points) over the 1-year course (= (-0.814) + 0.802; *p* = 0.020).

### Sensitivity analyses

**Fig. 4:**
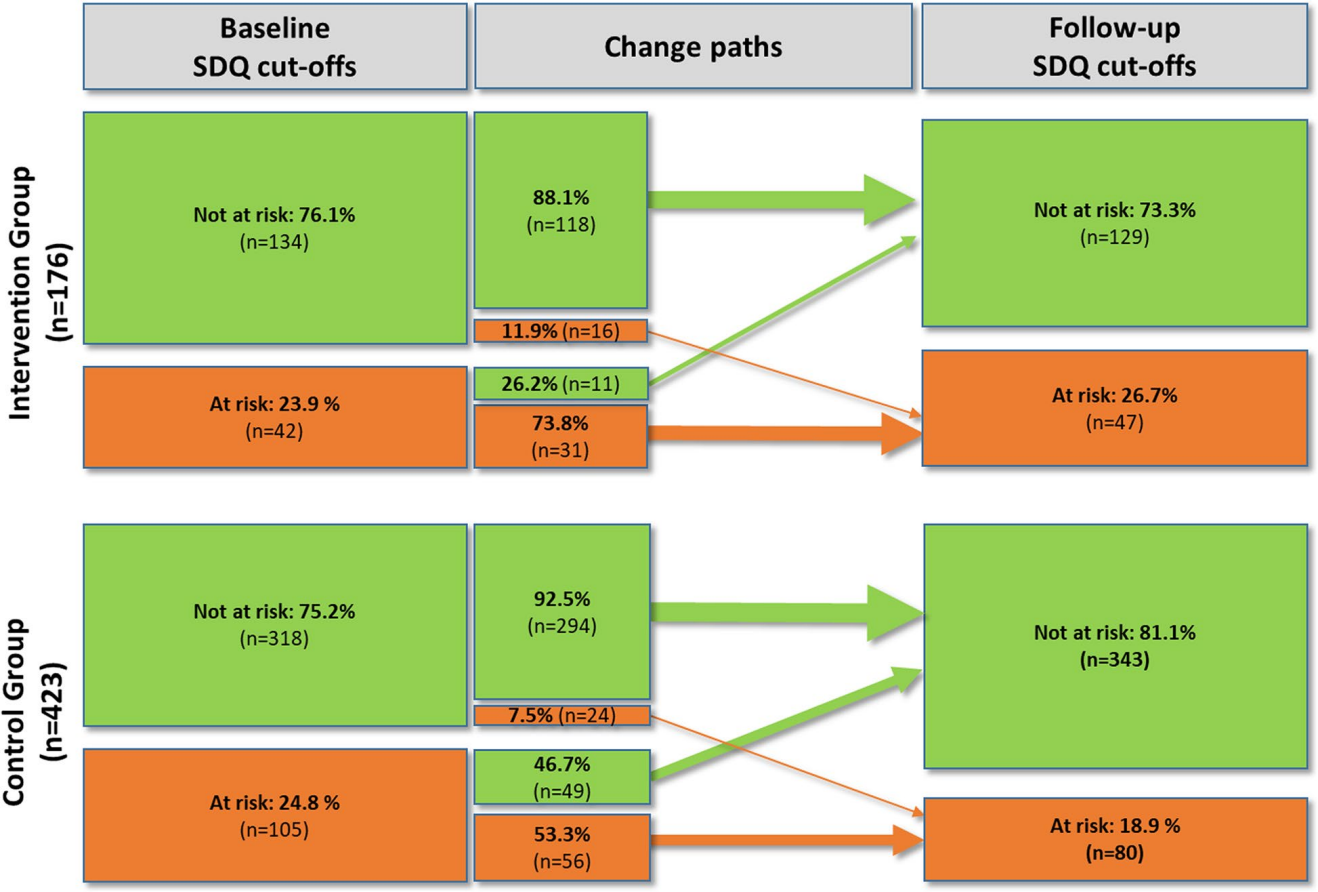
SDQ cut-offs at baseline as compared to follow-up in the HC and control group

In our sensitivity analyses, we modelled the change in SDQ cut-offs as shown in Fig. 4. At follow-up, 26.2% of the children in the HC (intervention) group and 46.7% of the control group showed an improvement in SDQ cut-offs (“at risk” at baseline and “not at risk” at follow-up). However, the vast majority showed no change (still at risk: 73.8 vs. 53.3%) and a sizeable proportion worsened (“not at risk” at baseline and “at risk” at follow-up: 11.9 vs. 7.5%). The change paths between the two groups were not significant (*p* = 0.056).

Also, we analysed the parental (SDQ-P) and the self-assessment version (SDQ-S) separately and compared the change in SDQ items (Supplementary Table 3). However, we could not find any significant differences between the two groups. There was a decrease in SDQ-S scores in boys (HC: -2.8, SD 4.1; control group:—2.2, SD 4.2), which was not statistically significant (*p* = 0.879). Using the different SDQ classifications yielded similar results compared to the total score. In contrast to using cut-offs, the age of the child was significantly associated with higher SDQ scores (0.161, *p* = 0.021) when introduced as a continuous variable.

A table presenting the characteristics of participants with missing follow-up can be found in the supplementary material (Supplementary Table 4). There were no significant health-related or demographic differences compared to participants with complete follow-up. Main stated reason in the lost to follow-up questionnaire was a lack of time.

## Discussion

In this cohort of children and adolescents with MHP, the SDQ scores were significantly associated with male sex and the educational level of the parents at baseline. In the follow-up period of 1 year, there was a significant improvement in SDQ scores in the control group while the scores in the intervention group remained stable.

The SDQ is a valid instrument to identify MHP in children and adolescents at an early stage. According to the SDQ authors, a minimal difference of more than 2 points in the SDQ total score is considered relevant. The deterioration in the control (-0.814) and intervention group (-0.012 points) over the 1-year course can therefore not be considered clinically relevant and suggests that the effect was too small to be detected. This could be attributed to 2 main reasons. Firstly, there was an average of 3 or 4 quarters delay between initial diagnosis and the online health assessment. This may have erased a possible significant effect of the HC programme on the child’s MHP developmental course. Another reason concerns the composition of the control group. Since the inclusion of participants could not be carried out via participating or non-participating paediatric practices, but via invitation letters by the health insurance, the selection had to take place via the billing data. It is therefore conceivable that paediatricians in the control group had completed the HC training but did not bill for the service. In this case, the training could change the behaviour of the paediatricians. This contamination is acceptable as it theoretically led to a more conservative result with a smaller difference between intervention and control group. When looking at participants with missing follow-up, there was no evidence that the two groups differed in terms of demographic or health characteristics that could have erased the effect in the intervention group. Ultimately, our results suggest that neither standard paediatric care nor HC training are effective in treating severe MHP.

In our population, the highest scores could be observed in boys and children with conduct disorder. This corresponds to the German literature, which reports significantly higher prevalence in boys (19.1%) than in girls (14.5%) [9]. One possible explanation is that conduct disorder becomes apparent earlier than emotional problems which are more common in girls. In contrast, a high or intermediate educational level of the parents was significantly associated with lower SDQ scores. This is also in line with the literature, since MHP are less pronounced in children with higher educated parents and a higher socioeconomic status, indicating that stress factors, coping skills and access to support measures are still unequally distributed socially [39, 41, 46].

Primary care paediatricians have decisive potential for early detection of MHP because of a high participation rate and acceptance of the routinely and periodically conducted developmental checks in children and adolescents [17, 47]. Due to the long care time, paediatricians have very good access to the family and can adequately assess the child’s need for support. This is consistent with the parents’ statements from the qualitative PrimA-QuO study [28]. Parents and adolescents reported satisfaction with the care provided in context of the HC programme. The families trusted their paediatrician even with more sensitive issues and remarked that their paediatrician allocated a large part of his consultation time to their problems. This was perceived as an indication of high quality of care. Quality of communication and an inclusion in the process of decision-making were also appreciated.

However, many paediatricians do not feel sufficiently trained to diagnose and treat MHP in primary care [18]. In the recent past, structured MHP programmes in paediatric care have been established to increase screening rates and treatment of MHP in primary care settings [20–23, 48, 49]. For example, a training programme in the Netherlands enabled primary care based general practitioners to identify more MHP than control practices [20, 49]. Additionally, the physicians were more reluctant to prescribe psychotropic drugs to children. Referral rates to mental healthcare remained relatively constant, but referrals shifted from specialised to primary mental healthcare. The question of whether improved screening leads to improved outcomes and better access to care has not yet been studied and reports for Germany remain scarce. Nevertheless, a variety of care services and integrative networking initiatives are available for children and adolescents with MHP. In Germany, measures for the prevention of mental disorders and the promotion of mental health have been initiated, for example in the form of projects in kindergartens and schools [38, 50, 51]. Since 2006, 2 additional developmental checks are offered for children between the ages of 7 and 11 to specifically examine behavioural problems, which allow MHP to be detected and treated at an earlier stage. This may also have contributed to the slight decline in MHP that could be observed in our study as well as in Germany in the last few years [9].

Our findings are particularly important as they generate the first evaluation of patient-related effects of the HC programme. In addition, our results will complement the qualitative PrimAQuO study [28] and the evaluation of costs of the HC programme [29] resulting in a comprehensive, mixed method programme evaluation. Programme evaluations are needed to make evidence-based decisions for the optimal care of children and adolescents with MHP in primary care.

The present study has several important strengths. The main strength of our study is an online health assessment of more than 1200 children with MHP and their parents. The children’s and adolescents’ health development could be followed over 1 year. We conducted a comprehensive SDQ assessment and were able to survey parents and the children themselves regarding their health development. We also obtained age-, gender- and indication-specific differences between intervention and control group.

Nevertheless, our study has some limitations. First, SDQ was only measured at 2 time points. We therefore had to model a linear association which might not reflect the true trajectories over time. Second, based on billing data, children meeting the inclusion criteria could be identified by the BKK with an average delay of 2 quarters, so that the baseline assessment took place months after the intervention. This may have erased an effect between both groups. Currently, the HC programme is limited to insured persons of the BKK funds. The BKK is a major statutory health insurance funds in Germany with 10.9 (in Bavaria: 2.4) of a total of 73.0 million insurees^6^ [52]. Therefore, the results of the present study are most likely to be generalisable for Germany. In addition, it has been shown in the international context that primary care programmes are likely to be integrable into different health system structures [20, 21]. How the corona pandemic — accompanied by school closures, discontinuation of school entry examinations and an increase in domestic violence — will affect the prevalence of MHP and care needs is still unknown.

## Conclusion

Our evaluation could not prove a clinically relevant effect of the HC programme on the developmental 1-year course of MHP among children and adolescents. Paediatricians provide low-threshold care and have crucial potential for early detection and treatment of mild MHP cases. Although neither the programme nor standard paediatric care showed significant improvements in MHP, the programme could be helpful in identifying MHP patients and choosing the best treatment option. Targeting families with low parental education might help reduce children’s and adolescents’ MHP and could be a step towards continuous improvement of care.

## Supplementary Information

**Additional file 1.** The additional file contains the in- and exclusion criteria of study participants, additional programme information, sensitivity analyses, information to the non-responder and lost to follow-up participants.

## Data Availability

The data that support the findings of this study are available from the corresponding author, (SD), upon request. All statistical analyses were carried out using SAS (Version 9.4, SAS Institute, Inc., Cary, NC, USA). The SAS codes can be provided on demand.

## Abbreviations

MHP: Mental health problems
HC: Health coaching
BKK-LV Bayern: Betriebskrankenkassen Landesverband Bayern (Bavarian State Association of company health insurance fund)
BVKJ: Berufsverband der Kinder- und Jugendärzte (a professional association of paediatricians in Germany)
SHI: Statutory health insurance
ICF: International Classification of Functioning, Disability and Health
ICD: International Statistical Classification of Diseases and Related Health Problems
SK: STARKE KIDS programme; with the SK, enhanced screenings for children and adolescents throughout Germany are available
SDQ: Strengths and Difficulties Questionnaire (SDQ-P/SDQ-S: parental/self-assessment version)

## Footnotes

1 The BKK contract working groups (VAG) of Bavaria, Baden-Württemberg and Hesse in cooperation with BVKJ and PaedNetz Bayern e.V. concluded a framework agreement according to the social security code (§ 140a et seq. SGB V in conjunction with § 43 para. 1 no.2 SGB V).

2 The contents of the HC training programme are not publicly accessible.

3 Further information on SHI services can be found in the literature on the corresponding fee schedule items "EBM 04,355" and "EBM 04,356" [25, 26].

4 The International Statistical Classification of Diseases and Related Health Problems (ICD) is a diagnostic classification standard for clinical and research purposes: “ICD has been the basis for comparable statistics on causes of mortality and morbidity between places and over time” [31].

5 More information regarding the SK and the HC programme is given here: https://www.bkkstarkekids.de/startseite/

6 Status as of 1.^st^ July 2019.
