# Supplementary material for: Longitudinal changes of mental health problems in children and adolescents treated in a primary care-based health-coaching programme – results of the PrimA-QuO cohort study
Source: BMC Prim Care. 2022 Aug 22;23:211. doi: 10.1186/s12875-022-01780-1 (PMC9396915; doi:10.1186/s12875-022-01780-1)
Supplement: Supplementary file 1 — Additional file 1. The additional file contains the in- and exclusion criteria of study participants, additional programme information, sensitivity analyses, information to the non-responder and lost to follow-up participants. [file 12875_2022_1780_MOESM1_ESM.pdf]

Additional File 1: Supplementary Information

to

Longitudinal changes of mental health problems in children and adolescents treated in a primary care-based health-coaching programme – results of the PrimA-QuO cohort study

Siona Decke[1,2*], Karina Hamacher[3], Martin Lang[4,5], Otto Laub[5], Lars Schwettmann[6,7], Ralf Strobl[1,8], Eva Grill[1,8*]

[1]Institute for Medical Information Processing, Biometry and Epidemiology - IBE, LMU Munich, Munich, Germany

[2]Pettenkofer School of Public Health, Munich, Germany

[3]BKK Vertragsarbeitsgemeinschaft Bayern, Munich, Germany

[4]Berufsverband der Kinder- und Jugendärzte (BVKJ) e.V., Cologne, Germany

[5]PaedNetz Bayern e.V., Munich, Germany

[6]Helmholtz Zentrum München – German Research Center for Environmental Health (GmbH), Institute of Health Economics and Health Care Management (IGM), Neuherberg, Germany

[7]Department of Economics, Martin Luther University Halle-Wittenberg, Halle (Saale), Germany

[8]German Centre for Vertigo and Balance Disorders, University Hospital, LMU Munich, Munich, Germany

*Corresponding author

Siona Decke, MPH

Email: siona.decke@med.uni-muenchen.de

Contents

Appendix A: The Health Coaching (HC) programme 1
Appendix B: Inclusion and exclusion criteria of study participants........ 3
Appendix C: Strengths and Difficulties Questionnaire (SDQ)........ 5
Appendix D: Sensitivity analyses 12
Appendix E: Non-Responder and lost to follow-up........ 14
References........ 17

Appendix A: The Health Coaching (HC) programme

In 2013, a major German statutory health insurance fund ("Betriebskrankenkassen Landesverband" - BKK-LV) in collaboration with a professional association of paediatricians ("Berufsverband der Kinder- und Jugendärzte" – BVKJ e. V.) has introduced a programme for their insurees targeted at primary care paediatricians (Health Coaching - HC). The foundation of the HC is the BKK programme "STARKE KIDS"[1] (SK). With the SK programme, additional developmental check-ups are available for children enrolled in the programme. In addition, the HC programme can be offered to children and adolescents with mental health problems (MHP) as shown in Supplementary Figure 1.

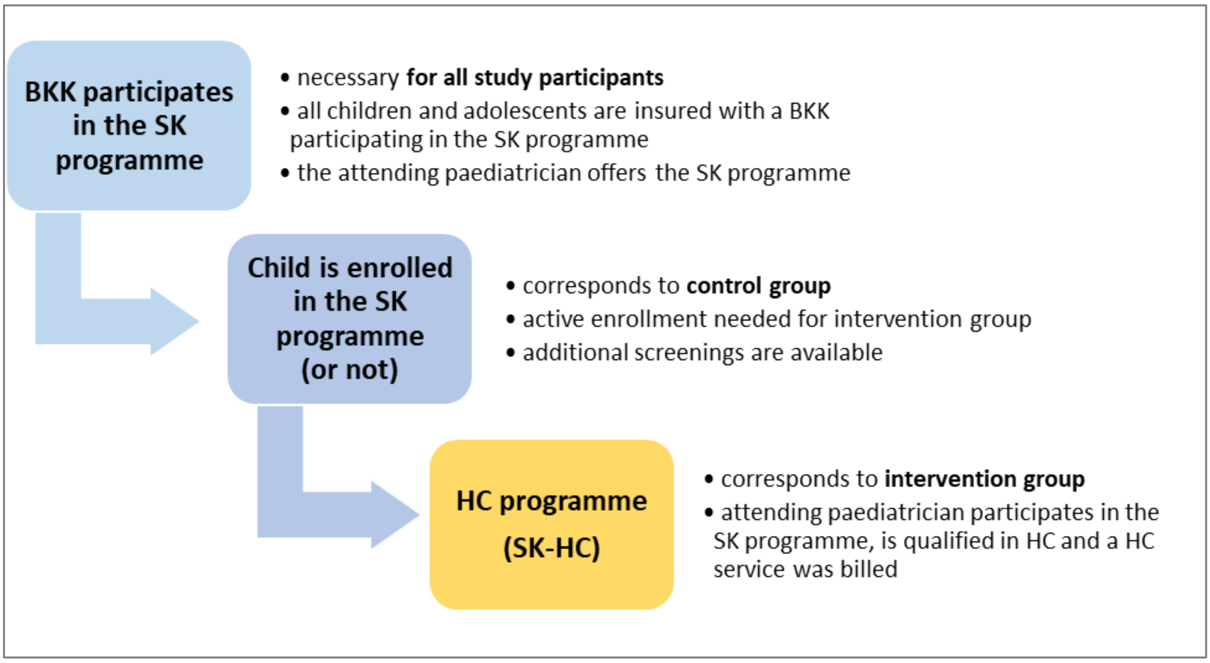

Supplementary Figure 1: Overview of the BKK STARKE KIDS (SK) and the Health Coaching (HC) programme

The HC is predominantly implemented in Bavaria, one of the largest federal states of Germany with a total of 13 million inhabitants. The HC has been available nationwide since October 2015. More than 700 paediatricians in Bavaria and more than 2.100 ones nationwide are currently qualified to participate in the HC programme and approximately 36.000 children with MHP have been treated accordingly to the programme. Paediatricians play a central role in this context. The aim of the patient education is to impart self-management skills to the children and their parents and purposefully inform them about the various care services available. The HC includes a training concept for paediatricians based on a dual training participation, standardised action guidelines for 16 MHP (e. g. developmental

[1] Further information can be found here: https://www.bkkstarkekids.de/unser-leistungsangebot/vorsorgeuntersuchungen-fuer-kinder-und-jugendliche

disorder of speech and language, enuresis, head and abdominal pain) and additional fees for paediatricians who undergo this specific training and demonstrably act according to the guidelines [1]. With the HC, 15 euros per 10 minutes up to a cap of 180 minutes per child in addition to the statutory standard care are billable. The basic programme's principles are participation, patient orientation and strengthening of existing resources. The social-paediatric diagnostic is divided into 3 steps as shown in Supplementary Figure 2.

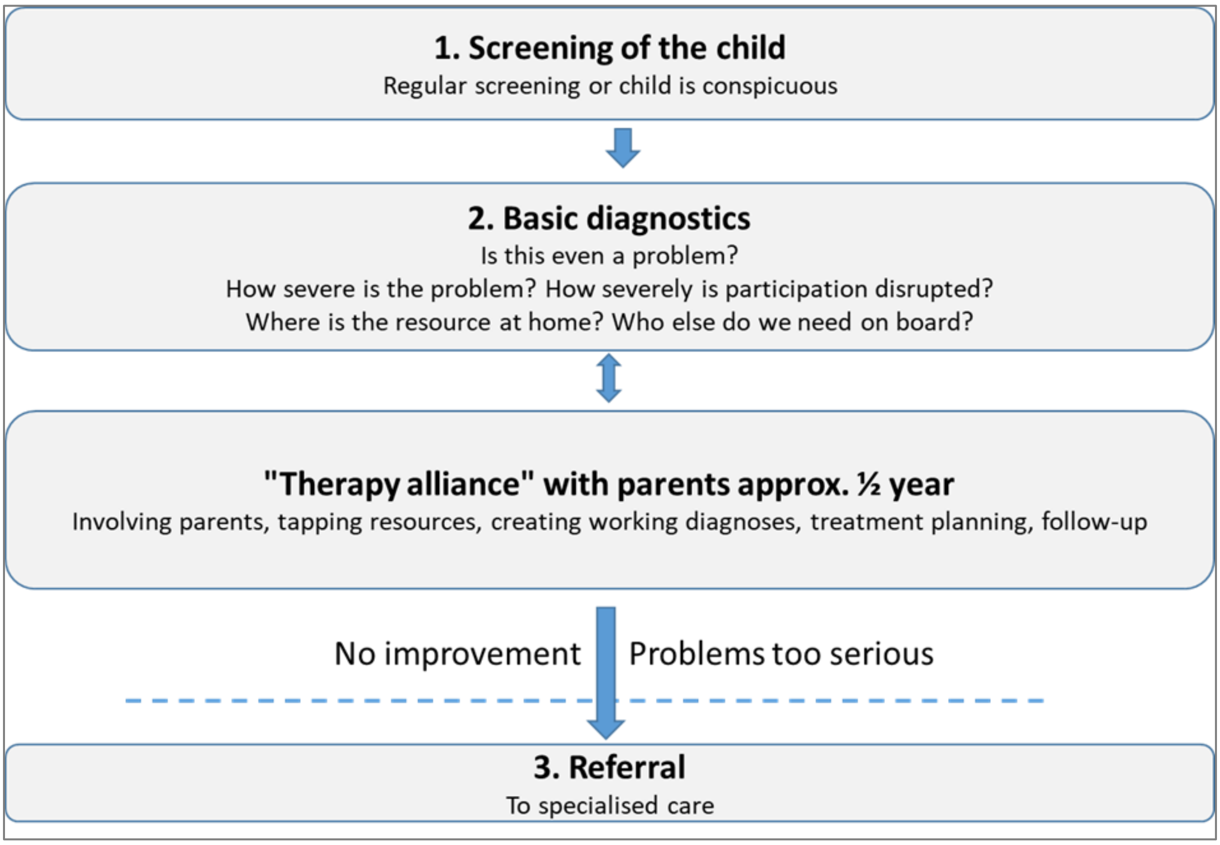

Supplementary Figure 2: 3 steps model of social-paediatric diagnostics

Typically, abnormalities are noticed at the developmental check-ups, by the parents themselves or at kindergarten/school. In a second step, the paediatrician carries out a detailed examination. Usually, a therapy alliance for 6 months is arranged with the parents, assessing the family's resources.

If the problems are too serious or the HC programme does not work, a referral to specialised care takes place. In this sense, children with minor problems can remain in the care of the paediatrician while resources are available for others who need immediate referral to specialised care (e.g., therapists, social paediatric centre). To implement the programme, it is necessary that the paediatrician participates in the SK programme and has completed the HC training participation and the child is enrolled in the SK programme so that HC services are billable.

Appendix B: Inclusion and exclusion criteria of study participants

Children and adolescents with mental health problems and their parents

Inclusion criteria:

- Children aged 0-17 years insured with a BKK that participates in the SK programme (prerequisite for querying the secondary data) and their parents
- Children have been diagnosed at least with 1 of the 4 most frequent MHP diagnoses:
    - developmental disorder of speech and language (ICD-10: F80.0-F80.9);
    - head and abdominal pain (somatoform) (ICD-10: G44.2, G43.0, G43.1, R10.4, F45.4);
    - conduct disorder (ICD-10: F68.8, F91.0-92.9, F94.0-95.9, F98.3-F98.9)
    - nonorganic enuresis (ICD-10: F98.0);
- Last paediatrician visit less than 6 months ago
- Intervention group:
    - enrolled in the BKK SK programme
    - attending paediatrician participates in the SK programme, was qualified in SK-HC and offered the SK-HC programme to the child
- Control group:
    - insured with a BKK that participates in the SK programme (enrolled or not)
    - attending paediatrician participates in the SK programme but did not offer the SK-HC programme to the child (qualified in HC or not)
- Signed informed consent for children aged 6 years and older
- Signed informed consent from parents

General exclusion criteria:

- Tentative diagnosis of MHP
- Insurance gap >30 days

In the Supplementary Figure 3 the intervention group and control group corresponding to the group definition above are illustrated.

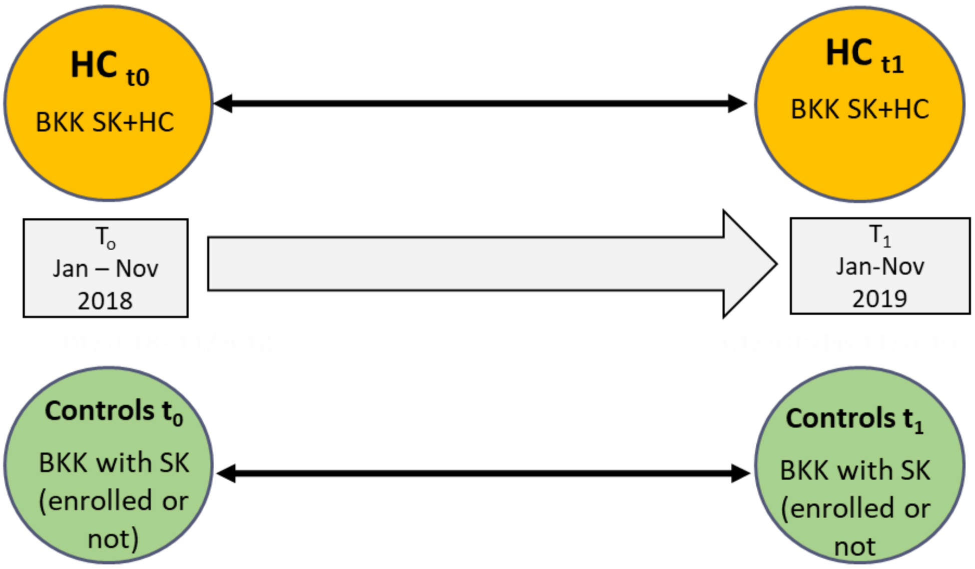

Supplementary Figure 3: Intervention (HC) and control group assessed at baseline (t0) and follow-up (t1) 1 year later.

SK=”STARKE KIDS” programme, HC=Health Coaching programme
t0=Baseline assessment, t1=Follow-up assessment 1 year later

Appendix C: Strengths and Difficulties Questionnaire (SDQ)

1. Overview SDQ

Children's mental health was assessed at baseline and follow-up using the German version of the Strengths and Difficulties Questionnaire (SDQ) [2,3]. The child and adolescent self-assessment version is used for children aged 11 years or older. A parental report is available for younger children. The SDQ comprises 25 items that contains 5 different sub-scales measuring (Supplementary Table 1):

Supplementary Table 1: Overview of the 5 SDQ sub-scales

**SDQ Total Score**	
1) emotional symptoms (5 items)	} 1) to 4) added together to generate a total difficulties score (based on 20 items)
2) conduct problems (5 items)	
3) hyperactivity/inattention (5 items)	
4) peer relationship problems (5 items)	
5) prosocial behaviour (5 items)	

Each of the SDQ items is scored on a 3-point scale with 0 = not true, 1 = somewhat true and 2 = certainly true, giving a maximum score of 10 for each sub-scale. Higher scores indicate more serious problems, except for prosocial behaviour, where higher scores indicate more positive behaviour. Following the SDQ scoring guide, these scores have been scaled up pro-rata if at least 3 items were completed for the sub-scale. If 2 or less items were completed, the sub-scale (and hence, the total SDQ) was treated as missing or incomplete. The questions for each sub-scale are the same at each age, with exception of 2 questions: The conduct disorder sub-scale varies slightly for 3-year-olds, as 2 items on antisocial behaviour are replaced by items on oppositionality.

The total difficulties score (range 0-40) is a sum of scores on 20 items (omitting prosocial items), with higher scores representing poorer psychosocial functioning. According to the German norms, a total parental assessment score ≤ 13 points is considered as "normal", a score of 14 up to 16 points as "borderline" and of 17 up to 40 points as "abnormal" (table 1). Congruently, in the self-assessment questionnaire the cut-offs are 0-15 ("normal"), 16-19 ("borderline") and 20-40 ("abnormal"). We used the more neutral terms "not at risk" (SDQ-P/SDQ-S: 0-13/0-15) and "at risk" (≥14/≥16). The German cut points for the SDQ total score and SDQ sub-scales are listed in Supplementary Table 2.

Supplementary Table 2: Overview of the german SDQ cut point for the total score and scores in the sub-scales

	Normal	Borderline	Abnormal
**Parental SDQ assessment**			
**Total difficulties score**	**0-13**	**14-16**	**17-40**
Emotional problems score	0-3	4	5-10
Conduct problems score	0-2	3	4-10
Hyperactivity score	0-5	6	7-10
Peer problems score	0-2	3	4-10
Prosocial score	6-10	5	0-4
Impact score	0	1	2-10
**SDQ self-assessment**			
**Total difficulties score**	**0-15**	**16-19**	**20-40**
Emotional problems score	0-5	6	7-10
Conduct problems score	0-3	4	5-10
Hyperactivity score	0-5	6	7-10
Peer problems score	0-3	4-5	6-10
Prosocial score	6-10	5	0-4
Impact score	0	1	2-10

In addition, the SDQ includes an impact supplement. When participants feel like having any emotional or behavioural difficulties (Variable: P102), additional questions follow. They concern duration (chronicity), suffering of the child to subjectively perceived social impairments in 4 areas of everyday life (at home, with friends, in the lessons at school/day care centre for preschool children, for leisure activities) and the impairment of the family. An impact score can be formed from the item "Difficulties upset or distress child" and the 4 items for everyday impairments are formed with the following scoring: 0= "not true, no, not at all", 1= "somewhat true, yes-minor, just a little" and 2= "certainly true, quite a lot". The items on overall distress and impairment can be summed to generate an impact score that ranges from 0 to 10 for parent- and self-report. An impact score of 2 or more points counts as conspicuous. At least 2 areas of life are impaired or in at least one area of life severe impairments due to mental health problems occur. Responses to the questions on chronicity and burden to others are not included in the impact score. When respondents negated the question if they feel like having any emotional or behavioural difficulties, they are not asked to complete the questions on resultant distress or impairment. In this case, the impact score is scored zero.

The SDQ follow-up versions include the 25 basic items and the impact question, but also 2 additional follow-up questions for use after an intervention (Has the intervention reduced any problems? Has the intervention helped in other ways, e.g. making the problems more bearable?).

2. SDQ: Generating scores in SAS

The SDQ scoring algorithm is based on 25 variables plus impact items for each questionnaire. The first letter of each variable name is 'p' for the parental SDQ version and 's' for the self-report SDQ assessment. After this first letter ('p' or 's'), the variable names are named as follows in the original scoring syntax e.g., "consid" is named as "**p**sconsid" in the parental scores and "**s**consid" in the self-assessment scores.

consid = Item 1 : considerate
restles = Item 2 : restless
somatic = Item 3 : somatic symptoms
shares = Item 4 : shares readily
tantrum = Item 5 : tempers
loner = Item 6 : solitary
obeys = Item 7 : obedient
worries = Item 8 : worries
caring = Item 9 : helpful if someone hurt
fidgety = Item 10 : fidgety
friend = Item 11 : has good friend
fights = Item 12 : fights or bullies
unhappy = Item 13 : unhappy
popular = Item 14 : generally liked
distrac = Item 15 : easily distracted
clingy = Item 16 : nervous in new situations
kind = Item 17 : kind to younger children
lies = Item 18 : lies or cheats *[for the SDQ for 2-4 year olds, replace 'lies' with 'argues']*
bullied = Item 19 : picked on or bullied
helpout = Item 20 : often volunteers
reflect = Item 21 : thinks before acting
steals = Item 22 : steals *[for the SDQ for 2-4 year olds, replace 'steals' with 'spite']*
oldbest = Item 23 : better with adults than with children
afraid = Item 24 : many fears
attends = Item 25 : good attention
ebddiff = Impact question: overall difficulties in at least one area
distres = Impact question: upset or distressed
imphome = Impact question: interferes with home life
impfrie = Impact question: interferes with friendships
impclas = Impact question: interferes with learning
impleis = Impact question: interferes with leisure

If the first response category (not true, no, not at all) has been selected, this is coded as "0", the next response category (somewhat true, yes-minor, just a little) is coded as "1" and the last category is coded as "2" (certainly true, quite a lot). The impact assessment has another category "3" (a great deal, yes-severe difficulties").

The algorithm generates 6 scores.

emotion = emotional symptoms (e.g. **p**emotion)
conduct = conduct problems
hyper = hyperactivity/inattention
peer = peer problems
prosoc = prosocial
ebdtot = total difficulties
impact = impact

Again, the first letter of each derived variable is 'p' for parent-based scores and 's' for self-report-based scores e.g., "emotion" is named as "**p**emotion" in the parental syntax and "**s**emotion" in the self-assessment.

*** P: PARENTAL SCORES**

```
if pconsid ge 0 then ppr1=1;
else ppr1=0;
if pshares ge 0 then ppr2=1;
else ppr2=0;
if pcaring ge 0 then ppr3=1;
else ppr3=0;
if pkind ge 0 then ppr4=1;
else ppr4=0;
if phelpout ge 0 then ppr5=1;
else ppr5=0;
pprotot=sum(pconsid,pshares,pcaring,pkind,phelpout);
pprono=ppr1+ppr2+ppr3+ppr4+ppr5;
pprosoc=round(pprotot*5/pprono,1);
if pprono lt 3 then pprosoc=.;
if ploner ge 0 then ppeer1=1;
else ppeer1=0;
if pfriend ge 0 then ppeer2=1;
else ppeer2=0;
if ppopular ge 0 then ppeer3=1;
else ppeer3=0;
if pbullied ge 0 then ppeer4=1;
else ppeer4=0;
if poldbest ge 0 then ppeer5=1;
else ppeer5=0;
pfrienx=2-pfriend;
ppopulax=2-ppopular;
ppeertot=sum(ploner,pfrienx,ppopulax,pbullied,poldbest);
ppeerno=ppeer1+ppeer2+ppeer3+ppeer4+ppeer5;
ppeer=round(ppeertot*5/ppeerno,1);
if ppeerno lt 3 then ppeer=.;
if prestles ge 0 then pha1=1;
else pha1=0;
```

```
if pfidgety ge 0 then pha2=1;
else pha2=0;
if pdistrac ge 0 then pha3=1;
else pha3=0;
if preflect ge 0 then pha4=1;
else pha4=0;
if pattends ge 0 then pha5=1;
else pha5=0;
preflecx=2-preflect;
pattendx=2-pattends;
phatot=sum(prestles,pfidgety,pdistrac,preflecx,pattendx);
phano=pha1+pha2+pha3+pha4+pha5;
phyper=round(phatot*5/phano,1);
if phano lt 3 then phyper=.;
if psomatic ge 0 then pem1=1;
else pem1=0;
if pworries ge 0 then pem2=1;
else pem2=0;
if punhappy ge 0 then pem3=1;
else pem3=0;
if pclingy ge 0 then pem4=1;
else pem4=0;
if pafraid ge 0 then pem5=1;
else pem5=0;
pemtot=sum(psomatic,pworries,punhappy,pclingy,pafraid);
pemno=pem1+pem2+pem3+pem4+pem5;
pemotion=round(pemtot*5/pemno,1);
if pemno lt 3 then pemotion=.;
if ptantrum ge 0 then pco1=1;
else pco1=0;
if pobeys ge 0 then pco2=1;
else pco2=0;
if pfights ge 0 then pco3=1;
else pco3=0;
if plies ge 0 then pco4=1;
else pco4=0;
if psteals ge 0 then pco5=1;
else pco5=0;
pobeyx=2-pobeys;
pcotot=sum(ptantrum,pobeyx,pfights,plies,psteals);
pcono=pco1+pco2+pco3+pco4+pco5;
pconduct=round(pcotot*5/pcono,1);
if pcono lt 3 then pconduct=.;
pebdtot=pemotion+pconduct+phyper+ppeer;
pdistrez=pdistres-1 ;
if pdistres=0 then pdistrez=0;
pimphomz=pimphome-1 ;
if pimphome=0 then pimphomz=0;
pimpfriz=pimpfrie-1 ;
if pimpfrie=0 then pimpfriz=0;
pimpclaz=pimpclas-1 ;
if pimpclas=0 then pimpclaz=0;
pimpleiz=pimpleis-1 ;
if pimpleis=0 then pimpleiz=0;
pimpact=sum(pdistrez,pimphomz,pimpfriz,pimpclaz,pimpleiz);
if pimpact lt 0 and pebddiff=0 then pimpact=0;
drop ppr1 ppr2 ppr3 ppr4 ppr5 pprotot pprono
ppeer1 ppeer2 ppeer3 ppeer4 ppeer5 pfrienx ppopulax ppeertot ppeerno
pha1 pha2 pha3 pha4 pha5 preflecx pattendx phatot phano
pem1 pem2 pem3 pem4 pem5 pemtot pemno
pco1 pco2 pco3 pco4 pco5 sobeyx pcotot pcono;
```

*** S: SELF-REPORT BASED SCORES**

```
if sconsid ge 0 then spr1=1;
else spr1=0;
if sshares ge 0 then spr2=1;
else spr2=0;
if scaring ge 0 then spr3=1;
else spr3=0;
if skind ge 0 then spr4=1;
else spr4=0;
if shelpout ge 0 then spr5=1;
else spr5=0;
sprotot=sum(sconsid,sshares,scaring,skind,shelpout);
sprono=spr1+spr2+spr3+spr4+spr5;
sprosoc=round(sprotot*5/sprono,1);
if sprono lt 3 then sprosoc=.;
if sloner ge 0 then speer1=1;
else speer1=0;
if sfriend ge 0 then speer2=1;
else speer2=0;
if spopular ge 0 then speer3=1;
else speer3=0;
if sbullied ge 0 then speer4=1;
else speer4=0;
if soldbest ge 0 then speer5=1;
else speer5=0;
sfrienx=2-sfriend;
spopulax=2-spopular;
speertot=sum(sloner,sfrienx,spopulax,sbullied,soldbest);
speerno=speer1+speer2+speer3+speer4+speer5;
speer=round(speertot*5/speerno,1);
if speerno lt 3 then speer=.;
if srestles ge 0 then sha1=1;
else sha1=0;
if sfidgety ge 0 then sha2=1;
else sha2=0;
if sdistrac ge 0 then sha3=1;
else sha3=0;
if sreflect ge 0 then sha4=1;
else sha4=0;
if sattends ge 0 then sha5=1;
else sha5=0;
sreflecx=2-sreflect;
sattendx=2-sattends;
shatot=sum(srestles,sfidgety,sdistrac,sreflecx,sattendx);
shano=sha1+sha2+sha3+sha4+sha5;
shyper=round(shatot*5/shano,1);
if shano lt 3 then shyper=.;
if ssomatic ge 0 then sem1=1;
else sem1=0;
if sworries ge 0 then sem2=1;
else sem2=0;
if sunhappy ge 0 then sem3=1;
else sem3=0;
if sclingy ge 0 then sem4=1;
else sem4=0;
if safraid ge 0 then sem5=1;
else sem5=0;
semtot=sum(ssomatic,sworries,sunhappy,sclingy,safraid);
semno=sem1+sem2+sem3+sem4+sem5;
semotion=round(semtot*5/semno,1);
if semno lt 3 then semotion=.;
if stantrum ge 0 then sco1=1;
else sco1=0;
```

```
if sobeys ge 0 then sco2=1;
else sco2=0;
if sfights ge 0 then sco3=1;
else sco3=0;
if slies ge 0 then sco4=1;
else sco4=0;
if ssteals ge 0 then sco5=1;
else sco5=0;
sobeyx=2-sobeys;
scotot=sum(stantrum,sobeyx,sfights,slies,ssteals);
scono=sco1+sco2+sco3+sco4+sco5;
sconduct=round(scotot*5/scono,1);
if scono lt 3 then sconduct=.;
sebdtot=semotion+sconduct+shyper+speer;
sdistrez=sdistres-1 ;
if sdistres=0 then sdistrez=0;
simphomz=simphome-1 ;
if simphome=0 then simphomz=0;
simpfriz=simpfrie-1 ;
if simpfrie=0 then simpfriz=0;
simpclaz=simpclas-1 ;
if simpclas=0 then simpclaz=0;
simpleiz=simpleis-1 ;
if simpleis=0 then simpleiz=0;
simpact=sum(sdistrez,simphomz,simpfriz,simpclaz,simpleiz);
if simpact lt 0 and sebddiff=0 then simpact=0;
drop spr1 spr2 spr3 spr4 spr5 sprotot sprono
speer1 speer2 speer3 speer4 speer5 sfrienx spopulax speertot speerno
sha1 sha2 sha3 sha4 sha5 sreflecx sattendx shatot shano
sem1 sem2 sem3 sem4 sem5 semtot semno
sco1 sco2 sco3 sco4 sco5 sobeyx scotot scono;
```

END OF SCORING SYNTAX

Appendix D: Sensitivity analyses

The individual change in SDQ total score (SDQ-P and SDQ-S combined) by intervention and control group was visualised as shown in Supplementary Figure 4. There were no remarkable changes detectable.

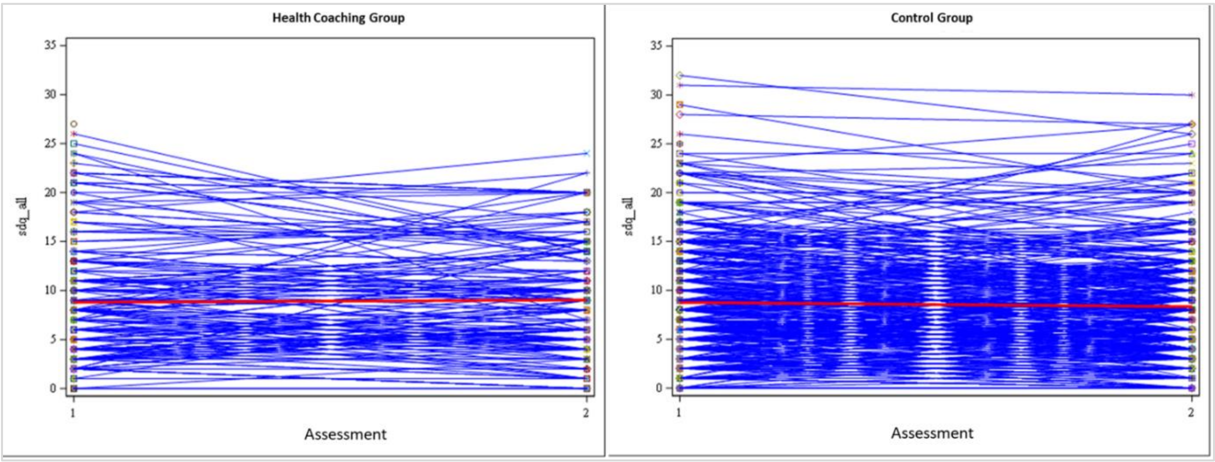

Supplementary Figure 4: Individual change in SDQ total score (SDQ-P and SDQ-S combined) by intervention and control group. The red line shows the average mean.

Both, the parental (SDQ-P) and the self-assessment version (SDQ-S) were analysed separately. The data are shown in Supplementary Table 3.

Supplementary Table 3: Change in the SDQ parental (SDQ-P) and self-assessment (SDQ-S) version during the follow-up

	Total	HC	Control	p-Value+
**Parental assessment**				
SDQ total score at follow-up* (SDQ-P)	8.4 (5.8)	9.0 (5.8)	8.2 (5.7)	0.074
Change in total score*	-0.4 (4.3)	0.0 (4.3)	-0.6 (4.1)	0.200
Change in boys*	-0.5 (4.3)	-0.2 (4.6)	-0.6 (4.2)	0.403
Change in girls*	-0.0 (3.7)	0.3 (3.7)	-0.2 (3.7)	0.365
Change in SDQ items (0-10)*				
emotional	-0.0 (1.6)	0.1 (1.7)	-0.1 (1.6)	0.382
conduct problems	-0.1 (1.5)	-0.1 (1.7)	-0.1 (1.5)	0.841
hyperactivity	-0.1 (1.8)	0.0 (1.8)	-0.2 (1.8)	0.421
peer relationship problems	-0.1 (1.4)	-0.0 (1.4)	-0.1 (1.4)	0.315
prosocial behaviour	0.3 (1.7)	0.2 (1.7)	0.3 (1.6)	0.667
Impact (yes)**	272 (53.2)	86 (55.8)	186 (52.2)	0.437
Change in impact	0.1 (1.1)	0.1 (1.1)	0.1 (1.2)	0.899
**Self-assessment of the child**				
Total score at follow-up* (SDQ-S)	10.0 (5.4)	11.1 (5.7)	9.7 (5.3)	0.448
Change in total score*	-1.2 (4.8)	-0.3 (5.6)	-1.5 (4.6)	0.292
Change in boys*	-2.2 (4.2)	-2.8 (4.1)	-2.0 (4.2)	0.879
Change in girls*	0.3 (5.4)	3.0 (5.9)	-0.7 (4.9)	0.088
Change in SDQ items (0-10)*				
emotional	-0.5 (2.1)	-0.4 (2.1)	-0.5 (2.2)	0.608
conduct problems	-0.4 (1.5)	-0.5 (1.6)	-0.4 (1.5)	0.751
hyperactivity	-0.5 (1.8)	-0.2 (2.1)	-0.6 (1.6)	0.690
peer relationship problems	0.2 (1.6)	0.8 (1.7)	0.0 (1.6)	0.090
prosocial behaviour	0.6 (1.8)	0.2 (1.9)	0.7 (1.8)	0.327
Impact (yes)**	53 (61.6)	15 (68.2)	38 (59.4)	0.464
Change in impact*	-0.3 (2.1)	-0.2 (1.2)	-0.3 (2.3)	0.837

* mean (standard deviation) ** n (%)
+ X2 test for categorical variables, Kruskal-Wallis-Test for continuous variables
SDQ parental assessment: n=514 (HC: 155/Control: 359)
SDQ self-assessment: n=88 (HC: 22/Control: 66l)

The overall change in SDQ-P between baseline and follow-up was -0.4 (SD 4.3, min -13.0, max: 15.0). The overall change in SDQ-S was -1.2 (SD 4.8, min -13.0, max: 5.0). There was a slight decrease in SDQ-S scores in boys (HC: -2.8, SD 4.1; Control group:- 2.2, SD 4.2), which was statistically not significant.

Appendix E: Non-Responder and lost to follow-up

1. Non-Responder

All participants who met the inclusion criteria were identified by the BKK based on the billing data. However, the response rate at baseline was low (17%). A second round of letters was therefore launched in 2019, but the response remained at 17%. In the pilot phase, patients who fulfilled the inclusion criteria were contacted via the paediatricians' practices, but the response rate was about 17 %, too. This may be due to the sensitive issue of mental health problems. Even though families were offered a small monetary compensation of 10 euros for their participation, each for the baseline and the follow-up survey, the response rate did not increase.

2. Lost to follow-up

491 (45 %) participants were lost to follow-up. Characteristics of participants with missing follow-up are shown in Supplementary Table 4.

Supplementary Table 4: Characteristics of participants with missing follow-up

	Total (n=1090)	With FU (n=599)	Lost to FU (n=491)	p-Value+
Responder (mother)	875 (87.9)	488 (87.6)	387 (88.4)	0.721
Children per family (n=430)				
1 child	73 (17.0)	34 (15.0)	39 (19.2)	0.499
2 children	267 (62.1)	145 (63.9)	122 (60.1)	
3 or more	90 (20.9)	48 (21.2)	42 (20.7)	
Age of the child in years*	6.9 (3.3)	6.7 (3.2)	7.1 (3.5)	0.038
Boys**	656 (60.2)	370 (61.8)	268 (58.3)	0.237
Age of the mother in years*	38.1 (5.2)	38.1 (5.0)	38.0 (5.4)	0.723
Age of the father in years*	41.0 (6.1)	41.0 (5.8)	41.1 (6.4)	0.888
Health of the parents (EQ5D)	0.9 (0.1)	0.9 (0.1)	0.9 (0.1)	0.590
VAS	84.6 (14.1)	84.9 (13.9)	84.2 (14.4)	0.520
Migrant background	175 (16.1)	90 (15.0)	85 (17.3)	0.306
Educational level of parents**				
high	562 (51.6)	341 (56.9)	221 (45.1)	<.0001
middle	424 (38.9)	227 (37.9)	197 (40.2)	
low	103 (9.5)	31 (5.2)	72 (14.7)	
Diagnosis of the child is known				
MHP diagnosis**				
head/abdominal pain	223 (20.5)	104 (17.4)	119 (24.2)	0.005
speech disorder	571 (52.4)	330 (55.1)	241 (49.1)	0.048
conduct disorder	262 (24.0)	145 (24.2)	117 (23.4)	0.884
enuresis	92 (8.4)	51 (8.5)	41 (8.4)	0.923
Parental assessment (n=922)				
SDQ score (0-40)*	8.4 (5.7)	8.7 (5.9)	8.1 (5.4)	0.189
SDQ score "at risk"**	214 (23.3)	127 (24.7)	87 (21.4)	0.241
Impact (yes)	411 (44.7)	242 (47.0)	169 (41.7)	0.111
Impact score (0-10)*	0.6 (1.3)	0.6 (1.4)	0.5 (1.2)	0.005
Self-assessment of the child (n=168)				
SDQ score (0-40)*	11.1 (6.4)	11.4 (6.3)	10.7 (6.5)	0.509
SDQ score “at risk”**	42 (25.0)	20 (23.8)	22 (26.2)	0.722
Impact (yes)	93 (55.4)	51 (60.7)	42 (50.0)	0.164
Impact score (0-10)*	1.2 (2.0)	1.2 (2.1)	1.2 (2.0)	0.775
Self-efficacy*(10-40)	27.8 (6.0)	27.7 (6.4)	27.9 (5.6)	0.855

* mean (standard deviation) ** n (%)
+ X2 test for categorical variables, Kruskal-Wallis-Test for continuous variables
n: Total= 1.090 (with FU= 599/ Lost to FU=491)
SDQ parental assessment: n=922 (with FU=515/ Lost to FU=407)
SDQ self-assessment: n=168 (with FU=84/ Lost to FU=84)

There were no significant health-related or demographic differences as compared to participants with complete follow-up (FU) that could explain the drop-out from the study. However, participants with 2

data collection points had slightly younger children on average (6.7 vs. 7.1 ($p=0.038$)), were more likely to belong to a higher educational level (56.9 vs. 45.1) and less likely to belong to a low educational level (5.2 vs. 14.7). Children with head and abdominal were more likely to drop out. No significant differences were found for the other diagnoses. This would indicate a short-lived diagnosis that possibly did not require further treatment. No differences were found between intervention and control group with regard to dropping out of the study.

In a next step, individual items from the family stress questionnaire (FABEL) and patient satisfaction (PACIC) were examined more closely. There was no indication that dissatisfaction or high satisfaction with medical care or high stress due to the child's diagnosis were reasons for dropping out either. A lost-to-follow-up questionnaire was provided. Main stated reason for non-participation was a lack of time.

References

1. Arbeitsgruppe Sozialpädiatrie des Berufsverbandes der Kinder- und Jugendärzte e.V. (BVKJ) (2016) BKK STARKE KIDS - Gesundheitsoaching. Handlungsleitfaden zur sozialpädiatrischen Sprechstunde für die Kinder- und Jugendärzte. AG Sozialpädiatrie BVKJ,
2. Goodman R (1997) The Strengths and Difficulties Questionnaire: a research note. The Journal of Child Psychology and Psychiatry 38 (5):581-586. doi:10.1111/j.1469-7610.1997.tb01545.x
3. Goodman A, Goodman R (2009) Strengths and difficulties questionnaire as a dimensional measure of child mental health. Journal of the American Academy of Child and Adolescent Psychiatry 48 (4):400-403. doi:10.1097/CHI.0b013e3181985068
